# Memory consolidation during sleep: a facilitator of new learning?

**DOI:** 10.1016/j.neuropsychologia.2025.109320

**Published:** 2025-11-17

**Authors:** Anna á V. Guttesen, Marcus O. Harrington, Melanie K. Fleming, M. Gareth Gaskell, Scott A. Cairney

**Affiliations:** a Department of Psychology, University of York, UK; b Oxford Centre for Integrative Neuroimaging, Nuffield Department of Clinical Neurosciences, University of Oxford, UK; c School of Psychology, University of East Anglia, UK; d York Biomedical Research Institute, University of York, UK

**Keywords:** Sleep, Systems memory consolidation, Next-day learning, Resource reallocation, Hippocampus

## Abstract

Sleep plays a crucial role in consolidating recently acquired memories and preparing the brain for learning new ones, but the relationship between these two processes is currently unclear. According to the prominent Active Systems Consolidation model, memory representations that are initially reliant on the hippocampus are redistributed to neocortex during sleep for long-term storage. An indirect assumption of this model is that sleep-associated memory processing paves the way for next-day learning by freeing up hippocampal encoding resources. In this review, we evaluate two central tenets of this ‘resource reallocation hypothesis’: (i) sleep-associated memory consolidation reduces hippocampal engagement during retrieval, and (ii) this reduction in hippocampal burden enhances the brain’s capacity for new learning. We then describe recent work that has directly tested the relationship between sleep-associated memory processing and next-day learning. In the absence of clear evidence supporting the resource reallocation hypothesis, we consider alternative accounts in which efficient learning is not contingent on prior overnight memory processing, but rather that sleep-associated consolidation and post-sleep learning rely on overlapping or independent mechanisms. We conclude by outlining how future research can rigorously test the resource reallocation hypothesis.

## 1 Introduction

Empirical work spanning an entire century has robustly demonstrated that sleep supports the consolidation of newly formed memories (Ashton and Cairney, 2021; Ashton et al., 2020; Ashton et al., 2022; Backhaus et al., 2008; Gais et al., 2006; Jenkins and Dallenbach, 1924; for a meta-analytic overview, see Berres and Erdfelder, 2021). More recent studies have also indicated that sleep supports next-day learning, potentially by restoring the brain networks that are central to encoding (Cousins et al., 2018; Guttesen et al., 2022; Mander et al., 2011; Ong et al., 2018; Poh and Chee, 2017; Van Der Werf et al., 2009; Vidal et al., 2025; Yoo et al., 2007). An emerging question from this collective body of research concerns the extent to which the benefits of sleep for memory consolidation and new learning are mechanistically linked. More specifically, does overnight consolidation lay the crucial groundwork for new learning? Or is it instead the case that sleep-associated memory strengthening and post-sleep learning rely on overlapping or independent mechanisms?

A prominent theory describing sleep’s role in memory retention is the Active Systems Consolidation (ASC) model (Brodt et al., 2023; Diekelmann and Born, 2010; Klinzing et al., 2019; Marshall and Born, 2007; Rasch and Born, 2013). This framework proposes that when an episodic memory is initially formed, its various components (such as the sights, sounds, and smells experienced while exploring an unfamiliar city) are encoded across distributed neocortical regions. Retrieval initially depends on the hippocampus, which binds the disparate neocortical representations into a cohesive episodic memory. During later non-rapid eye movement (NREM) sleep, coordinated memory reactivation events across hippocampus and neocortex are believed to gradually strengthen neocortical connections, until eventually the underlying memories become integrated within long-term knowledge and can be retrieved without involvement of the hippocampus. This hippocampal-neocortical dialogue is thought to be orchestrated by finely-tuned interactions between the three cardinal oscillations of NREM sleep: neocortical slow oscillations (SOs, <1 Hz), thalamocortical spindles (~12–15 Hz), and hippocampal ripples (~100–300 Hz), which coordinate the reactivation and reorganisation of newly formed memories in the sleeping brain (Staresina, 2024).

Since its conception, the ASC model has amassed considerable empirical support. There is evidence that NREM sleep oscillations and their close temporal coupling drive the overnight consolidation of recently acquired memories (Baena et al., 2020; Cox et al., 2012; Helfrich et al., 2018; Latchoumane et al., 2017; Leminen et al., 2017; Maingret et al., 2016; Mikutta et al., 2019; Mölle et al., 2011; Ngo et al., 2013; Ong et al., 2016; Prehn-Kristensen et al., 2020). Moreover, multiple lines of human and rodent research demonstrate that newly formed memories are repeatedly reactivated during sleep, with the magnitude of this reactivation predicting later retention (Cairney et al., 2018; Lee and Wilson, 2002; Schreiner et al., 2021). Finally, functional neuroimaging studies have shown that sleep prompts a gradual shift in the neural basis of episodic memory^2^ (Cairney et al., 2015; Gais et al., 2007; Samanta et al., 2021; Takashima et al., 2006), consistent with the hippocampal-neocortical dialogue.

The hippocampus is critically involved in episodic learning (Davachi and Wagner, 2002; Squire and Wixted, 2011). Given the evidence that sleep reduces hippocampal engagement during the retrieval of episodic memories acquired before sleep (Cairney et al., 2015; Samanta et al., 2021; Takashima et al., 2006), it is possible that overnight consolidation also increases hippocampal encoding capacity, supporting next-day learning (Walker, 2009). This is an indirect assumption of the ASC, which we refer to hereafter as the *resource reallocation hypothesis*. Recently, we addressed the question of whether sleep-associated memory processing and next-day learning are interconnected. Across two studies (Guttesen et al., 2022, 2025), measures of overnight memory retention and post-sleep learning were not significantly correlated in preregistered analyses. In the latter study, however, exploratory analyses controlling for baseline memory performance showed that overnight retention was correlated with next-day learning; a relationship that was not observed in a control group who remained awake during the consolidation interval. This preliminary evidence hints at a supporting role of sleep-associated memory processing in subsequent learning. Yet, the resource reallocation hypothesis has to date received little empirical attention.

In this narrative review, we first describe the resource reallocation hypothesis in detail, before reviewing the available evidence for two key tenets of this hypothesis: (i) sleep-associated memory consolidation reduces hippocampal engagement during later episodic memory retrieval, and (ii) this reduction in hippocampal burden enhances the brain’s capacity for new learning. We next consider an alternative, and perhaps more parsimonious, perspective: that learning is not actively facilitated by sleep-associated memory consolidation *per se*, but rather that memory consolidation and post-sleep learning are orchestrated by shared or distinct mechanisms. Finally, we recommend ways in which this critical gap in our understanding can be resolved in future research.

## 2 The resource reallocation hypothesis

**Fig. 1:**
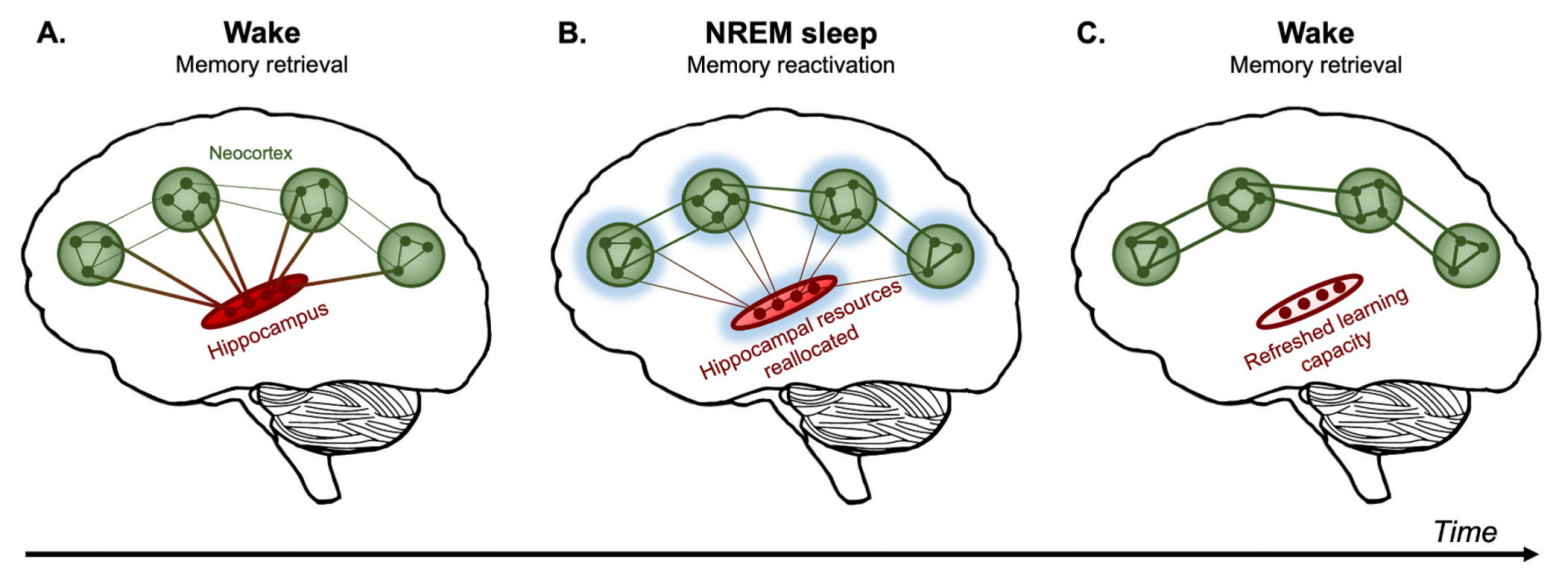
Schematic of the resource reallocation hypothesis. **(A)** When retrieving a recently encoded episodic memory during wake, the hippocampus binds disparate neocortical traces representing the memory’s various components. **(B)** During subsequent non-rapid eye movement (NREM) sleep, memory reactivations in the hippocampus and neocortex (depicted as a blue glow) strengthen cortico-cortical connections while weakening hippocampal-cortical connections. This process reduces the memory’s dependence on hippocampus, freeing up finite resources. **(C)** After sleep, hippocampal encoding capacity is restored, ensuring effective new learning. (For interpretation of the references to colour in this figure legend, the reader is referred to the Web version of this article.)

Evidence suggests that the hippocampus may have a finite capacity for information storage. During development and into adulthood, new neurons are continuously generated in hippocampal circuits (Ming and Song, 2005; Toni et al., 2007, 2008; Zhao et al., 2008). This phenomenon, known as neurogenesis, has repeatedly been shown to facilitate the acquisition of new hippocampal memories (Kempermann, 2022). However, neurogenesis also induces forgetting of other hippocampus-dependent memories (Akers et al., 2014; Scott et al., 2021). In their seminal study, Akers et al. (2014) found that increasing neurogenesis levels in mice led to accelerated forgetting of hippocampal memories, whereas reducing neurogenesis preserved older memories. These findings suggest that neurogenesis is an important mechanism of forgetting (Davis and Zhong, 2017), which may clear existing memory traces to free up resources for new learning. Along similar lines, the resource reallocation hypothesis proposes that the overnight consolidation of hippocampus-dependent memories alleviates the burden of maintaining older memories by freeing up finite cognitive resources and ultimately facilitating next-day learning (Fig. 1).

### 2.1 Sleep-associated memory consolidation reduces hippocampal burden

According to the resource reallocation hypothesis, sleep-associated consolidation is necessary to alleviate hippocampal burden and enhance the brain’s capacity for new learning. Neuroimaging studies have provided some evidence that sleep prompts such a shift in the neural basis of episodic memory. For example, Takashima et al. (2009) had participants encode face-location pairings before and after a 24-h delay, and then tested their memory for all of the pairs while they underwent functional magnetic resonance imaging (fMRI). Hippocampal activity was reduced for pairs encoded before the delay relative to those encoded afterwards, suggesting that overnight consolidation had reduced hippocampal engagement during retrieval.

According to the ASC model, systems consolidation occurs primarily during slow-wave sleep (SWS): the deepest stage of NREM sleep. Consistent with this view, sleep-associated changes in hippocampal engagement during retrieval have been linked to the amount of SWS obtained between learning and test. In their influential study, Takashima et al. (2006) trained participants on object-location pairings before a 90-min nap and then tested their memory for the pairs in the fMRI scanner. Individuals who spent more time in SWS during their nap exhibited better memory for the pairwise associations learned before-hand. Interestingly, longer SWS duration was also associated with reduced hippocampal engagement during memory retrieval. Along similar lines, a study in healthy older adults found that a greater magnitude of <1 Hz slow-wave activity (a characteristic oscillatory signature of SWS) was linked to less persistent hippocampal activation during post-sleep retrieval (Mander et al., 2015). Taken together, these findings suggest that SWS may contribute to hippocampal resource reallocation by facilitating overnight systems consolidation.

Importantly, however, other lines of research have offered a more nuanced perspective, suggesting that one or two nights of sleep may provide only marginal gains in hippocampal encoding resource (Gais et al., 2007; Joensen et al., 2024; Van Den Berg et al., 2022). For example, in Gais et al. (2007), participants learned a list of word pairs in the evening, before having their memory tested two days later. Between learning and test, participants either slept normally across both nights or were sleep deprived on the first night (obtaining recovery sleep on the second night). Word pair retrieval elicited stronger hippocampal responses when participants slept normally after learning relative to when they were sleep deprived. Interestingly, sleep after learning also increased functional connectivity between hippocampus and neocortex. Taken together, these results suggest that an initial period of sleep may be sufficient to promote systems-level changes in the neural basis of episodic memory (given the increase in hippocampal-neocortical connectivity), but insufficient to make any meaningful change in hippocampal encoding capacity. That said, the first night of sleep might be critical for resource reallocation in the longer term. In Gais et al. (2007), participants completed a surprise follow-up test six months later, which revealed that retrieval of words learned before sleep relied less on the hippocampus and more on the neocortex than retrieval of words learned before sleep deprivation, suggesting that post-encoding sleep may prompt longer-term changes in service of episodic learning. However, also note evidence demonstrating that the hippocampus may remain involved in memory retrieval after several weeks (Tambini et al., 2023), and even after a year (Vanasse et al., 2022).

Along similar lines, recent work has shown that hippocampal pattern completion can, in some cases, continue to support the holistic reinstatement of episodic memories following overnight consolidation (Joensen et al., 2024). Participants in this study learned three-element events in overlapping pairs (e.g., person-location, object-location, location-person) either immediately before or 24 h before a retrieval task that took place inside the fMRI scanner. Retrieval involved testing the learned pairs from each three-element event, with activity in non-target neocortical regions serving as an index of holistic reinstatement and activity in hippocampus acting as a proxy for hippocampal pattern completion. Interestingly, the data provided evidence of neocortical reinstatement that occurred with and without hippocampal pattern completion after 24 h, suggesting that the hippocampus and neocortex may support episodic memory retrieval through additive processes, at least in the first days after learning.

Because systems consolidation can occur more rapidly when to-belearned information is consistent with pre-existing cognitive schema (Tse et al., 2007, 2011), it is possible that prior knowledge might also permit more rapid resource reallocation during sleep. Interestingly, Hennies et al. (2016) found that sleep spindle density was negatively correlated with hippocampal engagement during next-day retrieval, but only when the newly learned memories were consistent with a pre-existing schema. Hippocampal burden might thus be reduced more quickly when memories consolidated during sleep are aligned with prior knowledge.

Some iterations of the ASC model argue that salient memories, such as those related to emotional or rewarding experiences, are preferentially consolidated during NREM sleep (Born and Wilhelm, 2012). If correct, emotionally salient memories should achieve hippocampal independence across sleep more swiftly than their emotionally neutral counterparts. Supporting this view, Cairney et al. (2015) found that SWS duration was predictive of better memory and reduced hippocampal engagement during the retrieval of emotionally negative images encoded before sleep, but not emotionally neutral or positive ones. Interestingly, another study observed contrasting findings, namely increased hippocampal activation during the retrieval of emotionally negative memories after sleep (Sterpenich et al., 2007). An important difference between these studies relates to the properties of the memories under investigation. Whereas Cairney et al. (2015) compared “remote” negative memories (encoded 24 h before retrieval) and “recent” negative memories (encoded immediately before retrieval), Sterpenich et al. (2007) compared remote negative and remote neutral memories. Hence, further work is needed to better understand how sleep contributes to resource reallocation in the context of emotionally salient information.

### 2.2 Sleep facilitates new hippocampus-dependent learning

If sleep-associated consolidation prepares the hippocampus for next-day learning, one would expect the acquisition of hippocampusdependent memories to be better after sleep than wakefulness. Consistent with this view, many studies have consistently shown improvements in episodic learning after sleep relative to sleep deprivation (Alberca-Reina et al., 2014; Cousins et al., 2018; Guttesen et al., 2022; Kaida et al., 2015; Poh and Chee, 2017; Saletin et al., 2016; Tempesta et al., 2016); a finding that is reliably supported by meta-analytic evidence (Crowley et al., 2024; Newbury et al., 2021). Findings from neuroimaging studies also align with the idea that sleep restores hippocampal learning mechanisms. Yoo et al. (2007) found that hippocampal activity during new learning was reduced among individuals who were sleep deprived, as compared to people who had obtained restful sleep. Importantly, the sleep-deprived participants also showed a significant deficit in retrieval performance two days later (after recovery sleep had taken place), suggesting that an absence of sleep had disrupted episodic encoding.

The neural oscillations supporting overnight consolidation may also set the scene for next-day learning. Consistent with this assumption, modifying slow oscillatory activity during SWS has been shown to impact later encoding of episodic memories. Van Der Werf et al. (2009) used sounds to disrupt overnight slow oscillatory activity (0.5–4 Hz) without reducing total sleep time and examined the effects of this manipulation on functional brain activity during a subsequent encoding task. Disrupted sleep (as compared to undisrupted sleep) reduced hippocampal engagement during learning and led to poorer performance in a later recognition test, supporting the view that slow sleep oscillations support the restoration of episodic learning networks.

Rather than disrupting slow oscillatory activity, Antonenko et al. (2013) used non-invasive electrical stimulation to enhance slow oscillations (~0.75 Hz) during NREM sleep. Boosting slow oscillatory activity improved the later learning of images, word pairs, and word lists, as compared to sham stimulation. In a similar study, Ong et al. (2018) enhanced slow oscillations with auditory closed-loop stimulation and found that the magnitude of this enhancement correlated with improved next-day learning and encoding-related activity in hippocampus. Together, these studies provide further evidence that slow sleep oscillations play a central role in restoring the brain’s capacity for new learning. It should be noted, however, that these studies only recruited young adults. More recent work performed in older adults found no effect of auditory stimulation on slow oscillation activity or new learning (Schneider et al., 2020). Further work is therefore needed to determine whether the relationship between sleep and next-day learning changes with age.

### 2.3 Does sleep-associated memory consolidation pave the way for new learning?

A central tenet of the resource reallocation hypothesis is that sleep-associated memory consolidation is a prerequisite for effective next-day learning. If correct, the amount of consolidation achieved during sleep should predict the capacity for new learning after sleep. We tested this hypothesis by examining the relationship between overnight memory retention and next-day learning in healthy young adults (Guttesen et al., 2022). Participants formed hippocampus-dependent declarative memories that were then consolidated across a night of sleep or total sleep deprivation, before learning a new set of hippocampus-dependent declarative memories the following morning. Sleep improved retention of the memories formed the previous evening, and enhanced learning of new memories formed the next morning, as compared to sleep deprivation. However, contrary to the resource reallocation hypothesis, there was no significant correlation between overnight memory retention and post-sleep learning. Moreover, in the sleep condition, slow oscillatory activity, which has been independently linked to both memory consolidation (Van Der Werf et al., 2009) and post-sleep learning (Antonenko et al., 2013), did not influence the relationship between these processes.

Considering these null results, one obvious possibility is that overnight memory consolidation does not support next-day learning, opposing the central tenet of the resource reallocation hypothesis. Alternatively, it is plausible that sleep-associated memory consolidation only facilitates post-sleep learning if the memories consolidated during sleep are qualitatively similar to the memories encoded after sleep. In the aforementioned study (Guttesen et al., 2022), overnight memory consolidation and next-day learning were evaluated using different hippocampus-dependent tasks: a visuospatial task and a paired associates task, respectively. To address this potential limitation, we conducted a follow-up study to examine whether consolidating one set of word pair memories across sleep correlates with the ability to learn a new set of word pair memories after sleep (Guttesen et al., 2025). Although our preregistered analyses again revealed no correlation between sleep-associated memory consolidation and post-sleep learning, a significant relationship was observed in exploratory analyses that controlled for individual differences in learning at baseline. This relationship was absent in participants who remained awake across the retention interval, suggesting that memory consolidation is only linked to subsequent learning when it takes place over sleep. Hence, these correlational findings offer preliminary support for the resource reallocation hypothesis.

## 3 Sleep’s role in consolidation and learning: overlapping or distinct mechanisms?

The resource reallocation hypothesis is grounded in research evidencing the role of NREM sleep oscillations in both consolidating memories and preparing the brain for new learning. However, direct empirical evidence supporting a relationship between overnight consolidation and next-day learning is currently limited (Guttesen et al., 2025). It is conceivable that, while the benefits of sleep for memory consolidation and new learning may be driven by overlapping neurocognitive operations, these processes do not have a direct causal relationship. In the following sections, we briefly explore the common or distinct mechanisms that could independently underpin overnight memory consolidation and optimised next-day learning.

### 3.1 Synaptic renormalisation facilitates memory consolidation and subsequent learning

The notion that offline consolidation and subsequent learning are facilitated by a shared underlying mechanism can be considered in the context of the Synaptic Homeostasis Hypothesis (SHY; Tononi and Cirelli, 2006, 2014). According to SHY, synapses are progressively potentiated due to experiential encoding during wakefulness, eventually reaching their saturation point by the end of the day. During SWS, slow oscillations drive a global downscaling of synapses, restoring them to their lowest levels by the time that sleep concludes. This process is thought to conserve space and energy within the brain, creating a sustainable environment for new learning.

The concept of synaptic potentiation during wakefulness followed by global renormalisation during sleep has amassed considerable (but not unequivocal) empirical support in animal models (Bushey et al., 2011; de Vivo et al., 2017; Gilestro et al., 2009; Liu et al., 2010; Spano et al., 2019; Vyazovskiy et al., 2008; also, see Durkin and Aton, 2016; Hengen et al., 2016). Evidence consistent with SHY has also been observed in humans. For example, cortical excitability increases with prolonged wakefulness and returns to baseline following sleep (Huber et al., 2013). Moreover, 4–8 Hz theta activity during wakefulness—an established neural marker of new learning (Köster et al., 2018; Osipova et al., 2006; Summerfield and Mangels, 2005)—is associated with increased slow oscillatory activity during subsequent sleep (Finelli et al., 2000; Huber et al., 2007), potentially reflecting a heightened need for synaptic renormalisation after intensive learning (however, see Grosmark et al., 2012; Watson et al., 2016, for evidence that synaptic renormalisation occurs during REM sleep).

Synaptic renormalisation in SWS is believed to support overnight consolidation by curtailing weak synaptic connections while sparing stronger ones, thereby increasing signal-to-noise ratios within neural networks engaged during prior learning (Tononi and Cirelli, 2006, 2014). By desaturating synaptic weights, SWS may simultaneously enhance the brain’s global capacity for new learning, offering an alternative account of how sleep-associated memory consolidation and next-day learning may be interrelated.

### 3.2 Slow brain rhythms drive consolidation and learning independently

Another perspective is that sleep underpins two distinct mechanisms: one that facilitates memory consolidation and another that optimises learning. These processes may rely on discrete components of 0.5–4 Hz slow oscillatory activity: slow oscillations (<1 Hz) and delta waves (1–4 Hz). While these rhythms have traditionally been treated as a single entity due to the difficulty of teasing them apart, innovative work suggests they may serve independent roles in memory processing. In their seminal study, Kim et al. (2019) used optogenetic stimulation to disrupt slow oscillations and delta waves separately in the rodent brain. Disrupting slow oscillations impaired motor memory retention, whereas disturbing delta waves improved it, suggesting that slow oscillations and delta waves play unique roles in consolidation and forgetting, respectively (Ngo and Born, 2019). Along the same lines, slow oscillations may support memory consolidation by driving the reactivation and redistribution of recently encoded memories, while delta waves may curtail weak synaptic connections, paving the way for next-day learning. Under this framework, sleep-associated memory consolidation and post-sleep learning are not causally dependent on one another but are instead driven by independent mechanisms with opposing functions.

## 4 Future directions

We have described an indirect assumption of the ASC model—the resource reallocation hypothesis—which explains how sleep facilitates memory consolidation whilst preparing the brain for new learning. In this section, we outline key outstanding questions and testable predictions of the resource reallocation hypothesis.

### 4.1 Does systems consolidation during sleep correlate with hippocampal engagement during post-sleep learning?

Evidence suggests that sleep reduces hippocampal engagement during later retrieval (Cairney et al., 2015; Takashima et al., 2006), but whether this reduction in hippocampal burden directly facilitates post-sleep learning has yet to be established. Future functional neuroimaging studies could investigate whether sleep-associated changes in hippocampal engagement at retrieval are linked to subsequent hippocampal activation during new learning. A relationship between these measures would provide support for the resource reallocation hypothesis. Such studies could employ hippocampus-dependent relational memory tasks, such as word-pair tasks or object-location tasks (Eichenbaum, 2004; Konkel and Cohen, 2009). To control for variability in baseline learning that may obscure the link between overnight consolidation and subsequent encoding (Guttesen et al., 2025), we recommend matching the strength of initial encoding across participants by training them to a fixed performance criterion. Furthermore, because hippocampal resource reallocation likely requires multiple nights of sleep (Gais et al., 2007), we recommend assessing performance across different delays. For example, in a multi-session study, one could compare hippocampal engagement during retrieval and encoding after one, two, or seven (or more) nights to better understand the evolution of systems consolidation and new learning.

### 4.2 Does manipulating sleep-associated memory consolidation affect post-sleep learning?

Extant work investigating the relationship between sleep-associated memory consolidation and post-sleep learning has relied on correlational designs (Guttesen et al., 2022, 2025). To draw causal conclusions, future studies could manipulate memory processing during sleep and observe its impact on post-sleep learning. One such approach would be to enhance overnight consolidation through non-invasive interventions that can boost NREM sleep oscillations, such as closed-loop auditory stimulation (Esfahani et al., 2023; Harrington and Cairney, 2021). Mediation analysis could test whether any benefit of auditory stimulation for learning is mediated by the impact of stimulation on sleep-associated memory consolidation.

### 4.3 Are sleep-associated memory consolidation and post-sleep learning supported by the same oscillatory phenomena?

The precise temporal coupling of slow oscillations and sleep spindles has been linked to overnight memory consolidation (Helfrich et al., 2018; Mikutta et al., 2019; Ong et al., 2016). Future rodent studies could optogenetically disrupt global slow oscillation-spindle coupling (Kim et al., 2019) and observe the effects on memory processing and new learning. If disruption impairs consolidation *and* post-sleep encoding, the results would be consistent with a shared sleep-associated mechanism. Conversely, if disruption impairs consolidation without affecting post-sleep encoding, the results would suggest that the benefits of sleep for learning and retention are driven by separate processes. Theoretically similar work in humans could investigate links between individual differences in slow oscillation-spindle coupling, sleep-associated memory consolidation, and next-day learning.

## Footnotes

2 Here, and throughout this article, we use the term *episodic memory* in line with much of the sleep and memory literature: to refer broadly to declarative learning tasks bound to a specific time and context (including, for example, word-pair recall paradigms and image recognition tasks). We acknowledge that some such tasks do not fully capture the phenomenological quality of episodic recollection, and that performance may sometimes be facilitated by processes besides mental re-experiencing.
